# Election of Officers of the Northern Medical Association

**Published:** 1852-03

**Authors:** J. Henry Smaltz

**Affiliations:** Sec. North. Med. Association


At a Stated Meeting of the Northern Medical Association held January 1st, 1852, the following members were elected as Officers for the present year.

*President.*—Dr. B. S. Janney.

*Vice President.*—Dr. N. L. Hatfield.

*Counsellors.*—Drs. J. F. Lamb, J. Uhler, J. Remington, G. W. Patterson, and J. N. Handy.

*Treasurer.*—Dr. M. B. Smith.

*Secretary.*—Dr. J. Henry Smaltz.

*Corresponding Secretary.*—Dr. J. Remington.

*Reporting Secretaries.*—Drs. H. Hartshorne, S. N. Troth.

*Delegates to American Medical Association.*—Drs. J. F. Lamb, R. H. Townsend, N. L. Hatfield, H. Hartshorne, W. Maybury and R. J. Levis.

Extract from the Minutes.

J. HENRY SMALTZ, Sec. North. Med. Association.

